# Efficacy and tolerability of fourteen-day sequential quadruple regimen: pantoprazole, bismuth, amoxicillin, metronidazole and or furazolidone as first-line therapy for eradication of *Helicobacter pylori*: a randomized, double-blind clinical trial

**DOI:** 10.17179/excli2019-1613

**Published:** 2019-08-19

**Authors:** Fariborz Mansour-Ghanaei, Alireza Samadi, Farahnaz Joukar, Hafez Tirgar Fakheri, Soheil Hassanipour, Mohammad Taghi Ashoobi, Soheil Soltanipour, Ahmad Alizadeh, Gholamreza Rezamand, Mohammad Fathalipour

**Affiliations:** 1 GI Cancer Screening and Prevention Research Center, Guilan University of Medical Sciences, Rasht, Iran; 2 Gastrointestinal and Liver Diseases Research Center, Guilan University of Medical Sciences, Rasht, Iran; 3 Caspian Digestive Disease Research Center, Guilan University of Medical Sciences, Rasht, Iran; 4 Gut and Liver Research Center, Mazandaran University of Medical Sciences, Sari, Iran; 5 Department of Pharmacology and Toxicology, Faculty of Pharmacy, Hormozgan University of Medical Sciences, Bandar Abbas, Iran

**Keywords:** Helicobacter pylori, eradication, furazolidone, sequential therapy, adverse effects

## Abstract

The optimal pharmacological regimen for eradication of *Helicobacter pylori* (*H. pylori*) has been investigated for many years. This study aimed to evaluate the efficacy and tolerability of bismuth-based quadruple therapy (B-QT) and a modified sequential therapy (ST) regimens in eradication of *H. pylori*. A randomized, double-blind trial was conducted on 344 patients. Patients with *H. pylori* infection and without a history of previous treatment were randomized to receive 14-day B-QT (bismuth subcitrate 240 mg, pantoprazole 40 mg, amoxicillin 1000 mg, and clarithromycin 500 mg twice daily) or 14-day ST (bismuth subcitrate 240 mg, pantoprazole 40 mg, amoxicillin 1000 mg, and metronidazole 500 mg twice a day for seven days followed by bismuth subcitrate 240 mg, pantoprazole 40 mg, amoxicillin 1000 mg, and furazolidone 100 mg twice a day for additional seven days). Drug adverse effects were assessed during the study. *H. pylori* eradication was determined eight weeks after the end of treatment using ^14^C-urea breath test. Based on per-protocol and intention-to-treat, the eradication rate was significantly higher (p<0.05) in the B-QT regimen 91.9 % (95 % CI; 88.1-94.0) and 90.2 % (95 % CI; 86.3-92.9), respectively compared to the ST regimen 80.8 % (95 % CI; 76.6-84.9) and 78.1 % (95 % CI; 73.7-82.4), respectively. The severity of vomiting and loss of appetite were significantly higher in ST regimen (p<0.05). The B-QT regimen was more effective and safer than the ST regimen. Conclusively, it is suggested to assess the efficacy and safety of this regimen in longer studies, larger population, and in other communities.

## Introduction

*Helicobacter pylori *(*H. pylori*), a gram-negative bacterium, infects about 50 % of industrialized nations and 80 % of developing countries population (Khademi et al., 2015[23]). *H. pylori* has a crucial role in the pathophysiology of acute and chronic gastritis, peptic ulcer, and gastric adenocarcinoma, and eradication of the bacterium is the main part of treatment of these conditions (Khademi et al., 2015[23]; Thung et al., 2016[39]). Drug efficacy and price, patient compliance, and bacterial resistance are the challenging factors in the pharmacotherapy of *H. pylori* (Fischbach and Evans, 2007[12]). 

Several studies have been carried out to evaluate the efficacy of current combination pharmacotherapies for *H. pylori* infection (Gisbert et al., 2000[19]; Fischbach et al., 2004[13]; Wang et al., 2017[41]). The most common regimens are a combination of a proton-pump inhibitor (PPI) and two antibiotics (triple therapy, TT), or co-administration of these agents with bismuth salts (bismuth-based quadruple therapy, B-QT) for 14 days (Malfertheiner et al., 2017[28]). Clarithromycin with amoxicillin or metronidazole are the frontline antimicrobial therapy for *H. pylori *eradication. However, resistant strains pose treatment failure and decreased efficacy of these regimens worldwide (Megraud, 2004[30]). Therefore, the antibiotics should be selected based on the local *H. pylori* resistances.

Evidences have shown that sequential therapy (ST), including a PPI and amoxicillin for the first five days followed by a TT including a PPI, clarithromycin, and metronidazole or tinidazole for five additional days, increases the efficacy of *H. pylori* eradication (Sanchez-Delgado et al., 2008[38]; Vaira et al., 2009[40]). Although, ST has demonstrated a satisfactory eradication rate (Gatta et al., 2009[15]), it has low patient compliance and facilitates the development of drug resistance (Yang and Sheu, 2011[43]).

The incidence of resistant* H. pylori* to metronidazole and clarithromycin is high among Iranian population (Ebrahimi-Dariani et al., 2003[4]; Salmanroghani et al., 2018[35]). Furazolidone-containing quadruple regimens achieved high *H. pylori* eradication among Iranian patients with or without previously failed treatment (Riahizadeh et al., 2010[34]; Ghadir et al., 2011[17]; Fakheri et al., 2012[6]). However, outcomes attributed to the substitution of furazolidone with metronidazole in ST have not been widely assessed.

The present study aimed to examine the efficacy and tolerability of B-QT (bismuth subcitrate, pantoprazole, amoxicillin, and clarithromycin for 14 days) regimen compared to a modified ST (bismuth subcitrate, pantoprazole, amoxicillin, and metronidazole for seven days followed by bismuth subcitrate, pantoprazole, amoxicillin, and furazolidone for additional seven days) regimen in eradication of *H. pylori* among patients referred to the endoscopy section of Razi Educational Center of Rasht, Iran.

## Materials and Methods

### Study design

This study was a prospective, randomized, double-blind controlled trial conducted between July 2017 and January 2018 at the Gastrointestinal and Liver Diseases Research Center (GLDRC) of Guilan University of Medical Sciences, in Rasht, Iran. All eligible participants signed an informed consent, and were randomly assigned to receive one of the assigned regimens. Patient compliance and the incidence of adverse drug reactions were determined after the end of treatment, and the eradication status was evaluated eight weeks after the end of treatment. The study was approved by the local ethics committee of the research center, and was carried out based on the declaration of Helsinki. The study was also reviewed and approved by the Iranian Clinical Trial Registry (IRCT201708071155N26).

### Patients

All patients attending to the endoscopy section of Razi Educational Center of Rasht, Iran were enrolled. Positive rapid urease test and/or positive histologic assessment of biopsies for *H. pylori* along with first diagnosis without previous treatment were considered as inclusion criteria. Patients with severe or unstable cardiovascular, hepatic, and renal diseases, history of allergy to any of the study medicines, hematological disorders, gastric and esophageal malignancy, and those who were pregnant or lactating were excluded from the study.

### Randomization and treatment

A block randomization method was used to ensure equal numbers in each groups of treatment. Patients were randomly allocated to receive a 14-day period of B-QT (bismuth subcitrate 240 mg, pantoprazole 40 mg, amoxicillin 1000 mg, and clarithromycin 500 mg twice daily) or a 14-day period of ST (bismuth subcitrate 240 mg, pantoprazole 40 mg, amoxicillin 1000 mg, and metronidazole 500 mg twice a day for seven days followed by bismuth subcitrate 240 mg, pantoprazole 40 mg, amoxicillin 1000 mg, and furazolidone 100 mg twice a day for additional seven days). It was recommended to patients that pantoprazole and bismuth subcitrate were taken before meals, and antibiotics after meals at the scheduled times. Demographic information including age, gender, and smoking status were recorded.

### Adverse effects and compliance evaluation

All participants were informed about potential adverse effects including bitter taste, nausea, vomiting, diarrhea, heartburn, abdominal discomfort, loss of appetite, headache, and dizziness. These adverse effects were evaluated during the study. To take >80 % of the prescribed medicines was considered as acceptable patient compliance.

### H. pylori eradication evaluation

Eight weeks after the completion of intervention, ^14^C-urea breath test (^14^C-UBT) was performed, based on our previous study (Mansour-Ghanaei et al., 2011[29]) to evaluate the treatment success. *H. pylori* eradication was considered as the result of <50 disintegrations per minute (DPM). Consumption of the medicines during these eight weeks were stopped to avoid interaction with ^14^C-UBT results.

### Statistical analysis

Both per-protocol (PP) and intention-to-treat (ITT) analyses were performed to determine the eradication rate of two regimens. Patients with poor compliance were excluded from the PP; however, ITT was conducted on all participants in the study including the patients that used the regimens out of the protocols or dropouts. *H. pylori* eradication rate, odds ratios and 95 % confidence interval were assessed for each group. To compare variables between two treatment groups, the *t* test was performed for quantitative variables and Fisher's exact test for qualitative variables. The results were expressed as mean ± SD (standard deviation) for quantitative variables, and the numbers and percentages for qualitative variables. SPSS version 18.0 (SPSS Inc., Chicago, IL, USA) was used to perform all analyses. A *P* value < 0.05 was considered statistically significant. 

## Results

### Characteristics of the patients

**Figure 1:**
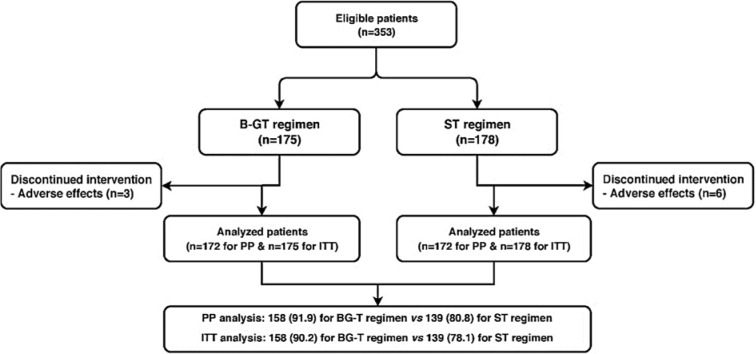
Flow chart for patient selection and randomization process B-QT; bismuth-based quadruple therapy, ST; sequential therapy, ITT; intention-to-treat, PP: per-protocol

A total of 353 participants with *H. pylori* infection were randomly assigned to either B-QT regimen (n=175) or ST regimen (n=178) groups. Three patients in the B-QT group and six patients in the ST group were excluded from the study due to low compliance, severe nausea, and severe anorexia. A flow chart of patient selection and randomization process is shown in Figure 1(Fig. 1). No statistically significant differences were observed between the two treatment groups in terms of age and gender. The number of smoker participants in ST group were higher that those of B-QT group. Demographic characteristics of the study patients is shown in Table 1(Tab. 1).

### Adverse effects and compliances 

Both B-QT and ST regimens were well-tolerated by a majority of the participants. The number of participants with adverse effects in the ST group (12.8 %) were higher compared to those of B-QT group (5.8 %). The most reported adverse effects were nausea and abdominal discomfort (Table 2(Tab. 2)). The compliance rates were 98.29 % and 96.93 % for the B-QT and ST groups, respectively. Three patients in the B-QT group and four patients in the ST group failed to ingest at least 80 % of the medications, and excluded from the PP analysis.

### Eradication rates

Based on both PP and ITT analyses, the eradication rate was significantly higher (p<0.05) in the B-QT regimen (Table 3(Tab. 3)). The eradication rates of B-QT and ST regimens were 91.9 % (95 % CI; 88.16-94.07) and 80.8 % (95 % CI; 76.6-84.9) in PP analysis, respectively. The eradication rates of B-QT and ST regimens were 90.2 % (95 % CI; 86.35-92.97) and 78.1 % (95 % CI; 73.7-82.4) in ITT analysis, respectively. There was no association between age groups, gender, and smoking status with eradiation rate for both groups in univariate analyses (P > 0.05 for all).

## Discussion

The results of the current study showed higher rates of *H. pylori* eradication with the 14-day B-QT regimen compared to the 14-day ST regimen among* H. pylori* positive patients who had no previous treatment. Both B-QT and ST regimens had good patient compliance, however, patients receiving B-QT regimen reported fewer adverse effects.

Both Canadian and European guidelines suggest to use triple therapy with a PPI, clarithromycin, and amoxicillin or metronidazole as first-line regimens in clinical practice (Malfertheiner et al., 2002[27]; Hunt et al., 2004[21]). However, the eradication success rate of these regimens is decreasing due to different pattern of *H. pylori* antimicrobial resistance (Yun et al., 2012[44]; Hsu and Peng, 2013[20]). Even less than 80 % of patients are infection eradicated with the most recommended quadruple therapy regimens (Jang et al., 2005[22]; Ghadir et al., 2011[17]). 

An increasing resistant infection to clarithromycin has been observed among Iranian patients (O'Morain and Montague, 2000[33]; Fakheri et al., 2018[8]). On the other hand, the cost of clarithromycin-containing regimens is relatively high in Iran. Hence, several other rescue regimens have been proposed in order to overcome these challenges (Malfertheiner et al., 2007[26]; Gisbert, 2012[18]; Kuo et al., 2012[25]). In one study, treatment with a seven-day regimen containing a PPI, bismuth subcitrate, amoxicillin, and high-dose of furazolidone (200 mg, twice daily) in patients with peptic ulcers and previous failed treatment achieved a *H. pylori* eradication rate of 69 % (Felga et al., 2008[11]). A similar seven-day rescue quadruple regimen containing a PPI, bismuth subcitrate, tetracycline, and a high-dose of furazolidone (200 mg, twice daily) obtained an eradication rate of 63 % (Eisig et al., 2005[5]). Another study conducted on the *H. pylori* eradication in the same reign of Iran used a sequential regimen containing a PPI, bismuth subcitrate, and amoxicillin along with high-dose of furazolidone (200 mg, twice daily) for the first five days, substituted by high-dose of metronidazole (500 mg, twice daily) for the following five days. The regimen demonstrated an eradication rate of 79.8 % (Mokhtare et al., 2015[31]).

Based on the findings of the present study, the B-QT regimen was successful in eradication of *H. pylori* in 91.9 % and 90.2 % of patients by PP and ITT analyses, respectively. The eradication rate of ST regimen was 80.8 % and 78.1 % of patients by PP and ITT analyses, respectively. Maastricht and other guidelines consider a cure eradication rate of > 80 % as an acceptable rate (Gao et al., 2010[14]). However, the ST regimen did not achieve the target threshold of an ideal regimen (eradication rate of > 90 %). It is likely that the low efficacy of the ST regimen used in the current study is related to the local pattern of antimicrobial resistance. The results of B-QT regimen are consistent with those of previous studies evaluating the efficacy of a ten-day quadruple therapy, comprised of a PPI, bismuth subcitrate, amoxicillin, and clarithromycin (twice daily). In that study, the eradication rate of *H. pylori* was 92.3 % (Wang et al., 2017[42]).

There was no relationship between demographic characteristics of patients including age groups, gender, and smoking status with the eradiation rate of both B-QT and ST regimens. Several previous studies have shown the eradication rates of anti-*H. pylori* regimens are not associated with age groups (Fakheri et al., 2004[7]; Chang et al., 2012[2]). Additionally, some other studies have demonstrated that the eradication success rate is not associated with smoking status (Chuah et al., 2012[3]; An et al., 2013[1]).

In addition to high efficacy and good compatibility with local microbial resistance patterns, an ideal regimen for *H. pylori* eradication should be well-tolerated, patient compatible, cost-effective, and easy to administer (Malfertheiner et al., 2002[27], 2007[26]). The compliance rates for the B-QT and ST regimen were 98.29 % and 96.93 %, respectively. Despite of the high compliance rate of both regimens, approximately one-third of patients experienced mild to moderate adverse events. The most reported adverse effects were nausea and abdominal discomfort. Generally, both B-QT and ST regimens were well-tolerated and patient compatible. Additionally, molecular studies particularly measuring the levels of *H. pylori* alongside with the effect of these recommended medicines are suggested (Samadani et al., 2016[36], 2019[37]; Fattahi et al., 2017[10], 2018[9]; Ghadami et al., 2019[16]; Kosari-Monfared et al., 2019[24]; Norollahi et al., 2019[32]). 

### Limitations

The absence of regional estimates of *H. pylori* eradication rates and local pattern of antimicrobial resistance are the most important limitation of the current study. Moreover, the results of the study may not be applicable to patients who had previous failed therapies, recurrence infections, or patients from other geographical part of Iran.

## Conclusion

In conclusion, the B-QT regimen provided higher eradication rates than the ST regimen. Moreover, the frequency of adverse effects in the B-QT regimen group were fewer. The B-QT regimen's medicines are also more available in different states of Iran. Thus, the ST regimen could not be an ideal alternative therapy for *H. pylori *eradication in this region. It is suggested to assess the efficacy and safety of this regimen in a longer studies, larger population, and in other communities.

## Notes

Fariborz Mansour-Ghanaei and Hafez Tirgar Fakheri (Gut and Liver Research Center, Mazandaran University of Medical Sciences, Sari, Iran; Telephone: +98-1315535116, Fax: +98-1315534951) contributed equally as corresponding authors. 

## Authors’ contribution

S.H, M.F and F.J analyzed data and drafted the article; A.S, F.M and H.T.F designed the protocol, enrolled participants, collected data, and edited the manuscript; F.J, G.R and A.S performed the culture and antimicrobial susceptibility test. A.S, M.T, S.S, A.A and H.T.F performed the gathering of clinical data on efficacy, compliance, and adverse events, and also managed the follow-up schedule; A.S and S.H provided protocol; S.H and M.F provided methodological advices about the culture and antimicrobial susceptibility test; F.M and M.F provided interpretation of the data and edited the manuscript; all authors have read and approved the final draft of the paper.

## Compliance with ethical standards

This study was conducted in compliance with the provisions of the Helsinki Declaration. The study was approved by ethics committee of Guilan University of Medical Sciences.

## Declaration of conflicting interests

The author(s) declare no potential conflicts of interest with respect to the research, authorship, and/or publication of the article.

## Funding

A substantial part of the study was supported by the Research Council of Guilan University of Medical Sciences.

## Acknowledgements

The assistance and cooperation of the Health Deputy at the Gastrointestinal and Liver Diseases Research Center, Guilan University of Medical Sciences who helped us in the data collection process are highly appreciated.

## Figures and Tables

**Table 1:**
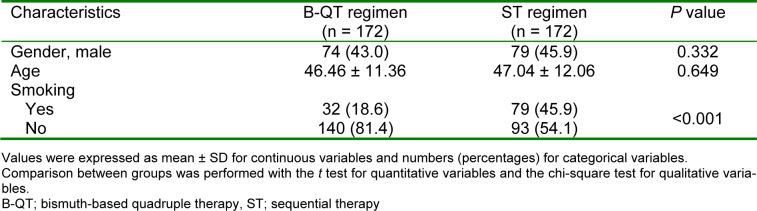
Demographic characteristics of the study patients

**Table 2:**
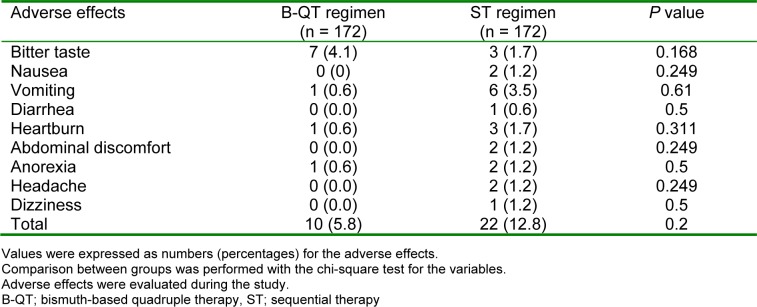
Adverse effects associated with the B-QT and ST regimen

**Table 3:**
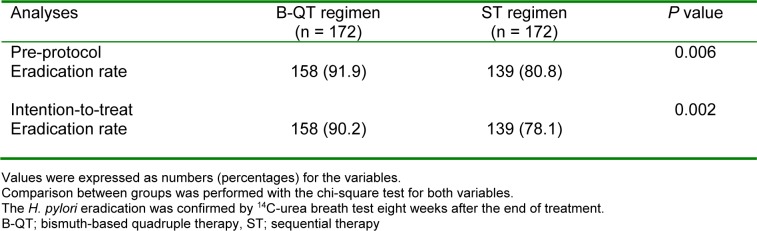
Eradication rates of the B-QT and ST regimen
